# Arterial graft with elastic layer structure grown from cells

**DOI:** 10.1038/s41598-017-00237-1

**Published:** 2017-03-10

**Authors:** Utako Yokoyama, Yuta Tonooka, Ryoma Koretake, Taisuke Akimoto, Yuki Gonda, Junichi Saito, Masanari Umemura, Takayuki Fujita, Shinya Sakuma, Fumihito Arai, Makoto Kaneko, Yoshihiro Ishikawa

**Affiliations:** 1 Cardiovascular Research Institute, Yokohama City University, Yokohama, Japan; 2 Department of Mechanical Engineering, Osaka University, Osaka, Japan; 3 Department of Neurosurgery, Yokohama City University, Yokohama, Japan; 4 Department of Micro-Nano Systems Engineering, Nagoya University, Nagoya, Japan

## Abstract

Shortage of autologous blood vessel sources and disadvantages of synthetic grafts have increased interest in the development of tissue-engineered vascular grafts. However, tunica media, which comprises layered elastic laminae, largely determines arterial elasticity, and is difficult to synthesize. Here, we describe a method for fabrication of arterial grafts with elastic layer structure from cultured human vascular SMCs by periodic exposure to extremely high hydrostatic pressure (HP) during repeated cell seeding. Repeated slow cycles (0.002 Hz) between 110 and 180 kPa increased stress-fiber polymerization and fibronectin fibrillogenesis on SMCs, which is required for elastic fiber formation. To fabricate arterial grafts, seeding of rat vascular SMCs and exposure to the periodic HP were repeated alternatively ten times. The obtained medial grafts were highly elastic and tensile rupture strength was 1451 ± 159 mmHg, in which elastic fibers were abundantly formed. The patch medial grafts were sutured at the rat aorta and found to be completely patent and endothelialized after 2.5 months, although tubular medial constructs implanted in rats as interpositional aortic grafts withstood arterial blood pressure only in early acute phase. This novel organized self-assembly method would enable mass production of scaffold-free arterial grafts *in vitro* and have potential therapeutic applications for cardiovascular diseases.

## Introduction

Biological tissue-engineered blood vessels, which possess elasticity and withstand arterial blood pressure, have considerable potential to improve the outcome for patients with cardiovascular disease. Small-caliber artificial grafts are especially desirable, due to the limited availability of healthy autologous vessels in the elderly population with coronary heart disease, peripheral artery obstruction, or severe renal disease^1^. Biological arterial grafts or sheets also offer considerable benefit to patients with congenital heart disease, due to their growth potential. There have been many studies aimed at fabrication of biological tissue-engineered blood vessels with adequate mechanical strength, elasticity, and growth potential. Although a recent study demonstrated implantable scaffold-free tubular graft composed of multicellular spheroids^2^, a method for the fabrication of artificial scaffold-free elastic arterial media has not yet been established. This is partially because it is difficult to generate the elastic structure of arterial media, i.e., multiple layers of helically arranged smooth muscle cells (SMCs) surrounded by elastic fiber components. This structure plays a key role in determining vascular elasticity.

We previously demonstrated that arterial media-mimetic constructs, consisting of either human umbilical arterial SMCs (hUASMCs) or rat neonatal aortic SMCs, were grown in layered cell culture^3, 4^. However, when they were pulled under tension, they were readily ruptured, indicating that elastic layer structure was incomplete and that tensile strength was not sufficient. It is well known that vascular SMCs, during tissue development, are continuously exposed to mechanical pressure stress, and that such mechanical stress plays a role, at least in part, in developing the vascular tissue components. We thus thought that hydrostatic pressurization might promote the maturation of layered vascular SMCs. In support of this idea, hydrostatic pressure has been reported to correlate to synthesis of extracellular matrix (ECM) proteins, such as collagens, elastin, and sulfated glycosaminoglycans^5–7^.

## Results

### Periodic hydrostatic pressurization promoted stress fiber formation and fibronectin fibrillogenesis in vascular SMCs

**Figure 1:**
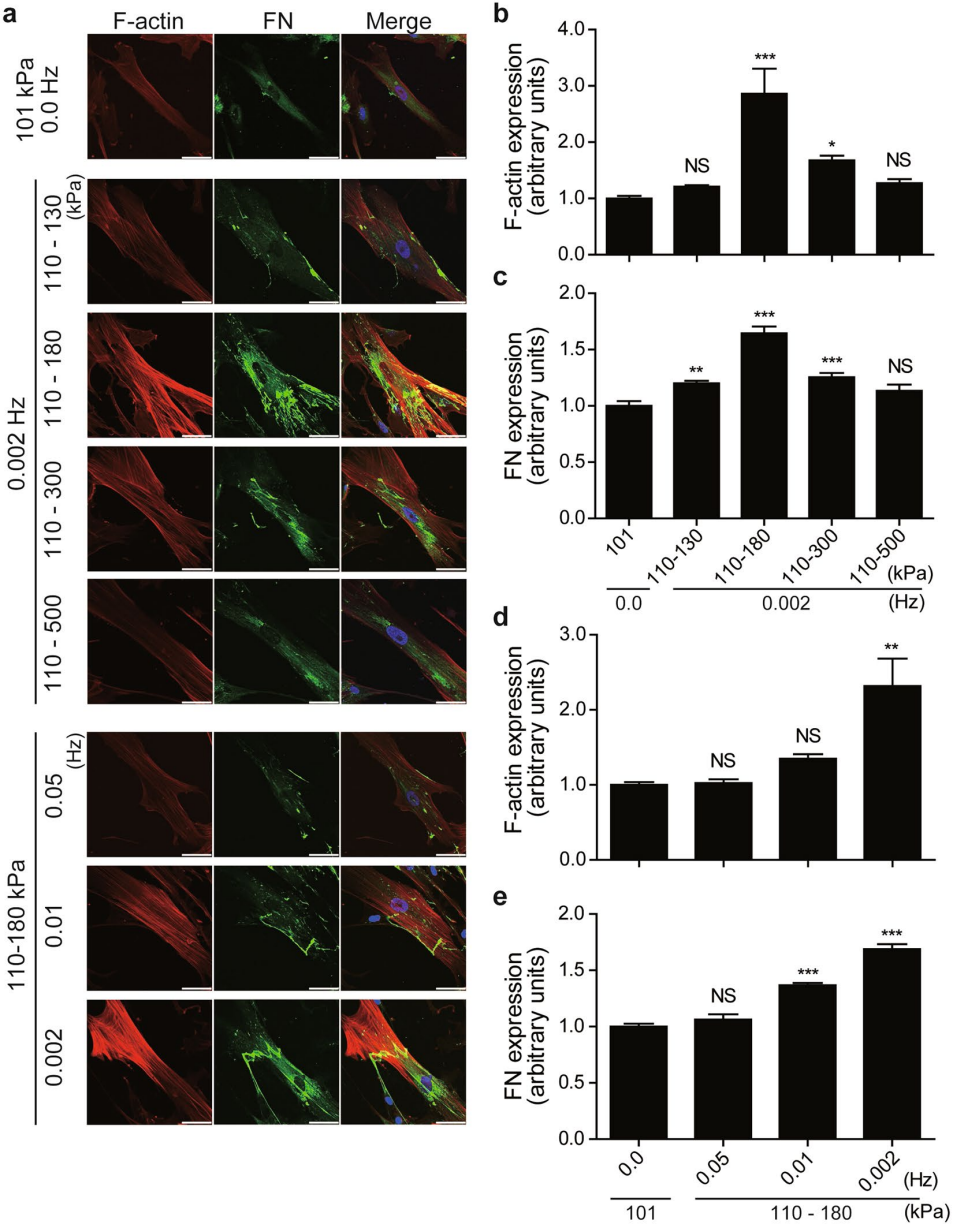
The effects of periodic hydrostatic pressurization (PHP) on stress-fiber formation and fibronectin (FN) fibrillogenesis. (**a**) Representative images of immunocytochemistry of hUASMCs exposed to various patterns of PHP for 24 h. Cells were stained for F-actin and FN to visualize stress-fiber formation and FN fibrillogenesis. Scale bars: 50 μm. (**b** and **c**) Quantification of intensity of F-actin and FN of the cells exposed to pressure from 130 kPa to 500 kPa in which lower pressure was kept at 110 kPa and frequency was maintained at 0.002 Hz. (**d** and **e**) Quantification of intensity of F-actin and FN of the cells exposed to pressure from 0.05 Hz to 0.002 Hz in which lower and higher pressure was kept at 110 kPa and 180 kPa, respectively. n = 9–16, NS, not significant vs. 101 kPa, 0.0 Hz (atmospheric pressure); *p < 0.05; **p < 0.01; ***p < 0.001. Values are shown as the mean ± standard error of the mean (SEM) of independent experiments. Data were analyzed using analysis of variance (ANOVA) followed by Dunnett’s multiple comparison test.

First, we examined the effect of various patterns of periodic hydrostatic pressurization (PHP) on stress fiber formation and fibronectin (FN) fibrillogenesis in human umbilical artery smooth muscle cells (hUASMCs) using a custom-made pressurization system (Supplemental Fig. 1), because FN fibrillogenesis is the critical step for several scaffold ECMs and organogenesis^8^. When hUASMCs were exposed to various degrees of pressurization at 0.002 Hz for 24 h, the setting of PHP 110 to 180 kPa (equivalent to 65–590 mmHg) significantly increased stress fiber formation and FN fibrillogenesis; these changes were not observed in hUASMCs cultured under 101 kPa (atmospheric pressure) (Fig. 1a–c). When the lower pressure was kept at 101 kPa instead of 110 kPa, the viability of cells was reduced, and stress fiber formation and FN fibrillogenesis were not enhanced (data not shown). Next, we examined the effect of the frequency of PHP, and found that 0.002 Hz was a better condition for stress fiber formation and FN fibrillogenesis than 0.05 Hz and 0.01 Hz (Fig. 1a,d and e). Because the cells were plated on uncoated glass coverslips, these data suggested that certain settings of PHP induce fibrillogenesis of SMC-derived FN. When cells were exposed to a cycle slower than 0.002 Hz, levels of pH could not be adjusted to a stable 7.4, even if the CO_2_ concentration was changed. Therefore, we utilized 110 to 180 kPa and 0.002 Hz of PHP for further studies.

**Figure 2:**
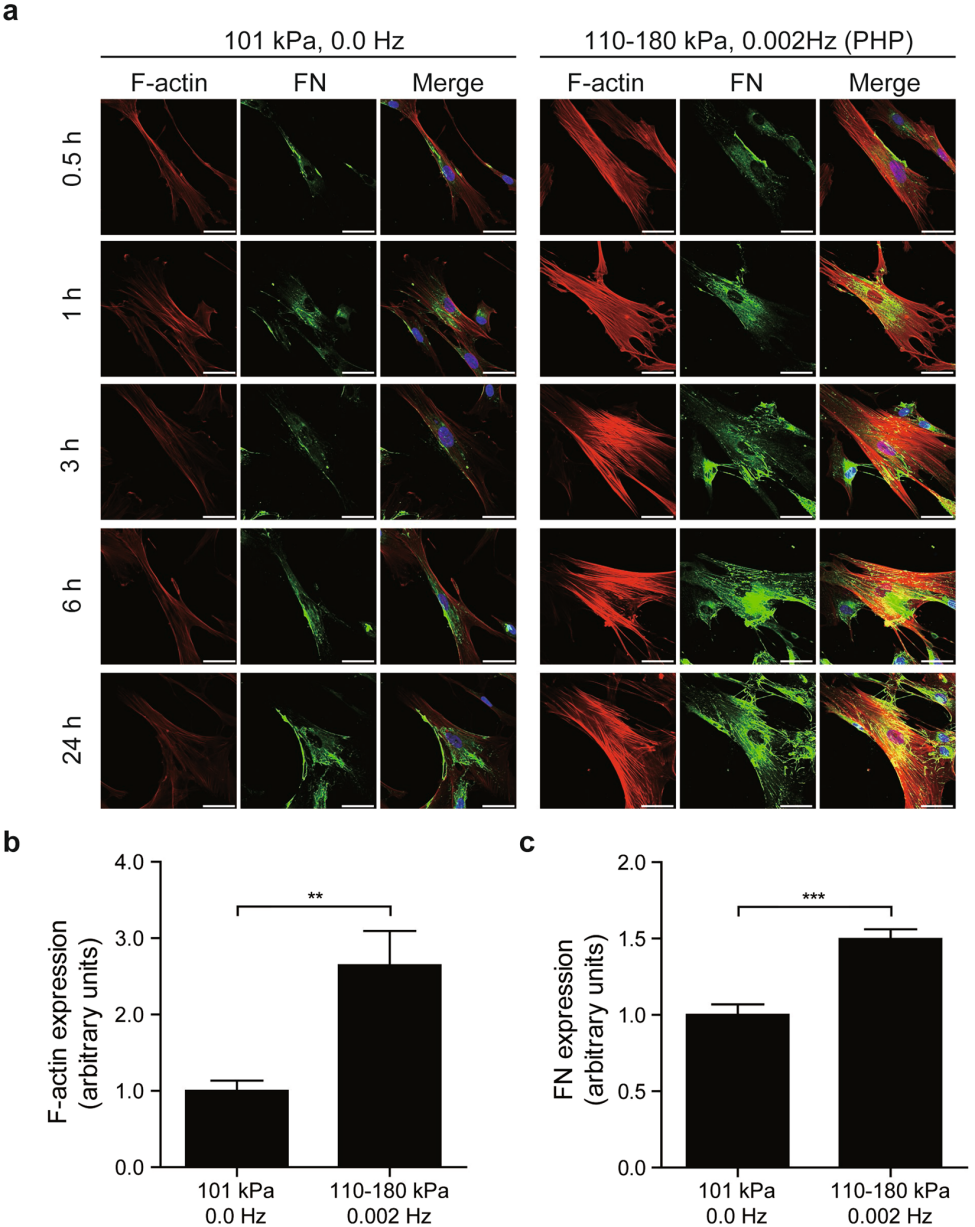
Time-dependent effect of PHP pressurization on stress-fiber formation and FN fibrillogenesis. (**a**) Representative images of immunocytochemistry of hUASMCs exposed to PHP (110–180 kPa, 0.002 Hz) up to 24 h. Cells were stained for F-actin and FN to visualize stress-fiber formation and FN fibrillogenesis. Scale bars: 50 μm. (**b** and **c**) Quantification of intensity of F-actin and FN of cells exposed to the pressure for 24 h, respectively. n = 11–12, **p < 0.01; ***p < 0.001 vs. 101 kPa, 0.0 Hz (atmospheric pressure). Values are shown as the mean ± standard error of the mean (SEM) of independent experiments. Data were analyzed using two-tailed unpaired Student’s *t*-test.

Next, we investigated the time-course effect of PHP (110–180 kPa, 0.002 Hz) in hUASMCs. Thirty minutes after PHP, stress fiber formation became detectable, unlike that in cells cultured under 101 kPa (Fig. 2a). Fibrillogenesis of FN was observed 1 h after PHP (Fig. 2a). Significant changes in stress fiber formation and FN fibrillogenesis were observed 24 h after PHP (Fig. 2b,c). These data suggested that the supraphysiological setting of PHP induced FN fibrillogenesis.

### Rho/ROCK pathway mediated the PHP-induced stress fiber formation and FN fibrillogenesis

**Figure 3:**
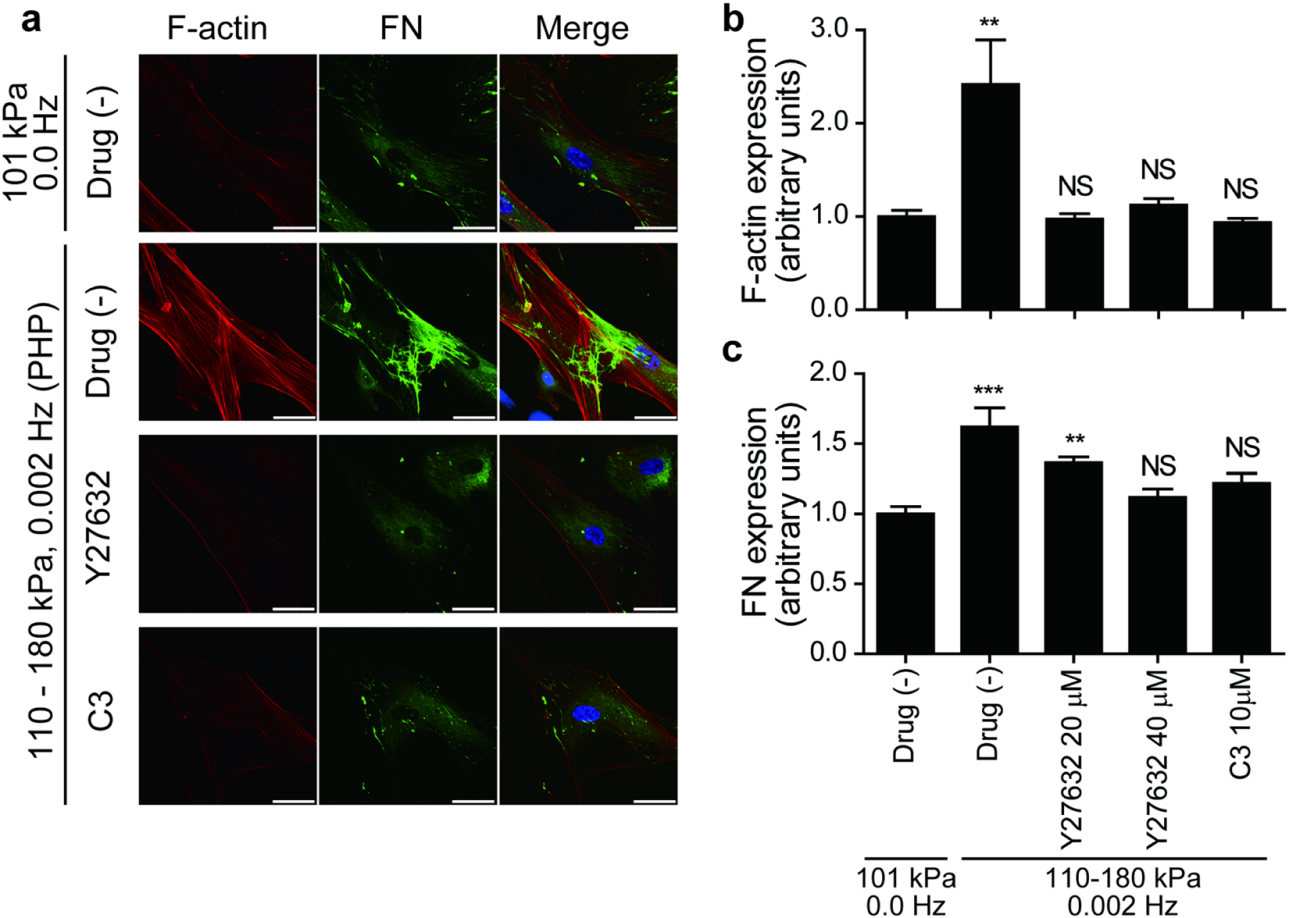
The effect of Rho kinase inhibition on stress-fiber formation and FN fibrillogenesis. (**a**) Representative images of immunocytochemistry of hUASMCs exposed to PHP (110–180 kPa, 0.002 Hz) for 24 h. Cells were incubated with Y27630 (40 μmol/L) or C3 (10 μmol/L) during pressurization and stained for F-actin and FN. Scale bars: 50 μm. (**b** and **c**) Quantification of intensity of F-actin and FN of (**a**), respectively. n = 7–11, NS, not significant vs. 101 kPa, 0.0 Hz (atmospheric pressure); **p < 0.01; ***p < 0.001. Values are shown as the mean ± standard error of the mean (SEM) of independent experiments. Data were analyzed using analysis of variance (ANOVA) followed by Dunnett’s multiple comparison test.

Since it is known that mechanical stress, including hydrostatic pressure, stimulates Rho/ROCK signaling pathways^9^, we investigated the involvement of the Rho/ROCK pathway in PHP-induced stress fiber formation and FN fibrillogenesis using a Rho kinase inhibitor C3 and a ROCK inhibitor Y27632. Preincubation of hUASMCs with C3 or Y27632 significantly attenuated PHP (110–180 kPa, 0.002 Hz, 24 h)-induced stress fiber formation and FN fibrillogenesis (Fig. 3).

### The effect of PHP on gene expression

**Figure 4:**
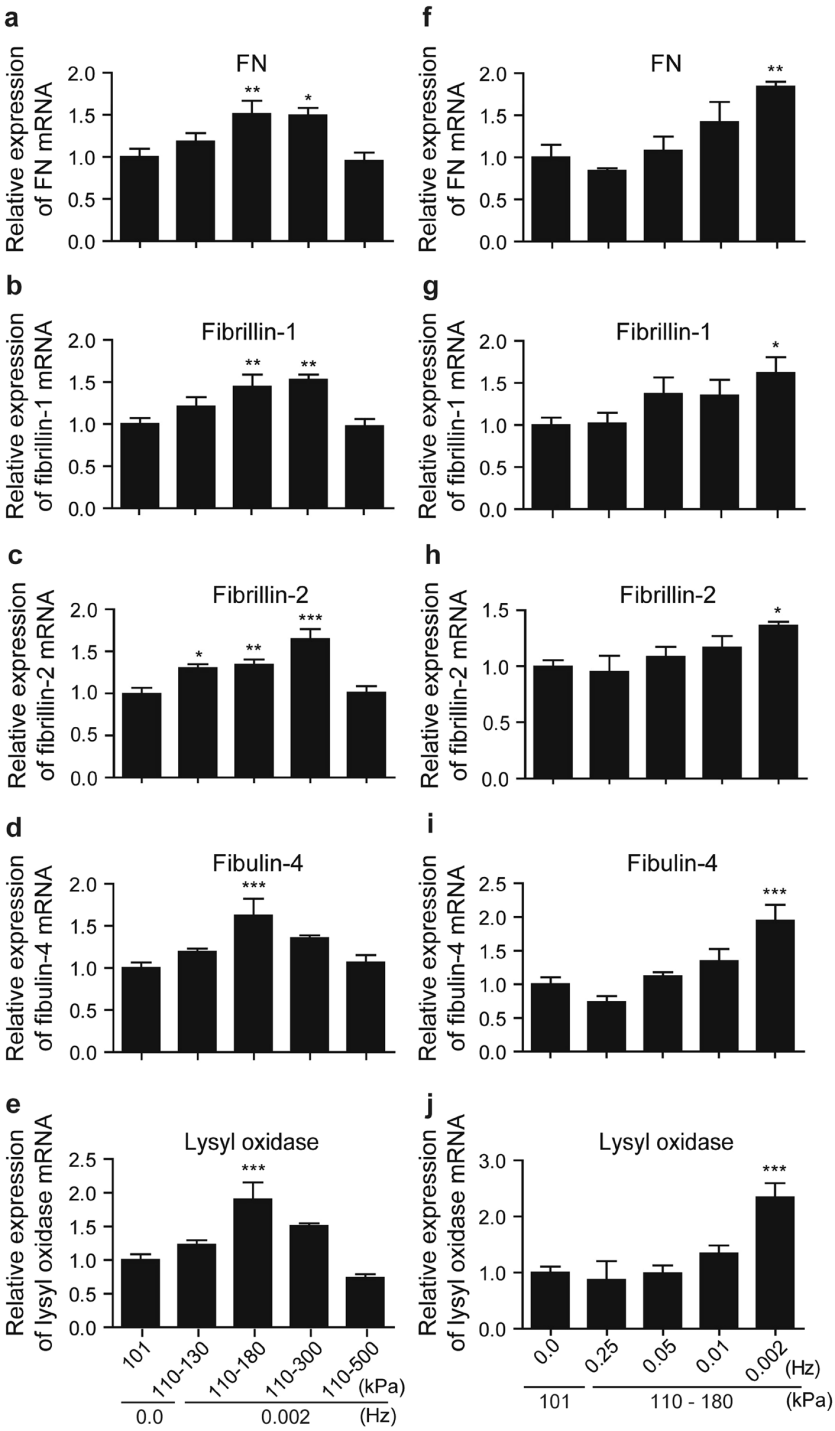
PHP-induced gene expression. (**a**–**e**) Suspensions of hUASMCs were exposed to pressure from 130 kPa to 500 kPa in which lower pressure was kept at 110 kPa and frequency was maintained at 0.002 Hz. Expression levels of FN, fibrillin-1, fibrillin-2, fibulin-4, and lysyl oxidase mRNAs were quantified by real-time RT-PCR. (**f**–**j**) Suspensions of hUASMCs were exposed to pressure from 0.25 Hz to 0.002 Hz in which lower and higher pressure was kept at 110 kPa and 180 kPa, respectively. Genes were quantified in the same way as (**a**–**e**). n = 4–7, NS, not significant vs. 101 kPa, 0.0 Hz (atmospheric pressure); *p < 0.05; **p < 0.01; ***p < 0.001. Values are shown as the mean ± standard error of the mean (SEM) of independent experiments. Data were analyzed using analysis of variance (ANOVA) followed by Dunnett’s multiple comparison test.

Elastic fiber formation and collagen production are critical to the development of tunica media. We next examined the mRNA expression of elastic fiber-related genes and collagens in SMCs exposed to various settings of PHP for 18 h. In accordance with the immunocytochemistry data (Figs 1 and 2), PHP with 110 to 180 kPa at 0.002 Hz significantly increased the levels of FN, fibrillin-1, fibrillin-2, fibulin-4, and lysyl oxidase mRNAs (Fig. 4). Messenger RNA expression levels of tropoelastin, fibulin-5, and types I, III, and IV of collagens did not show a clear tendency induced by various settings of PHP (Supplemental Fig. 2).

### Fabrication of the layered structure of vascular SMCs

**Figure 5:**
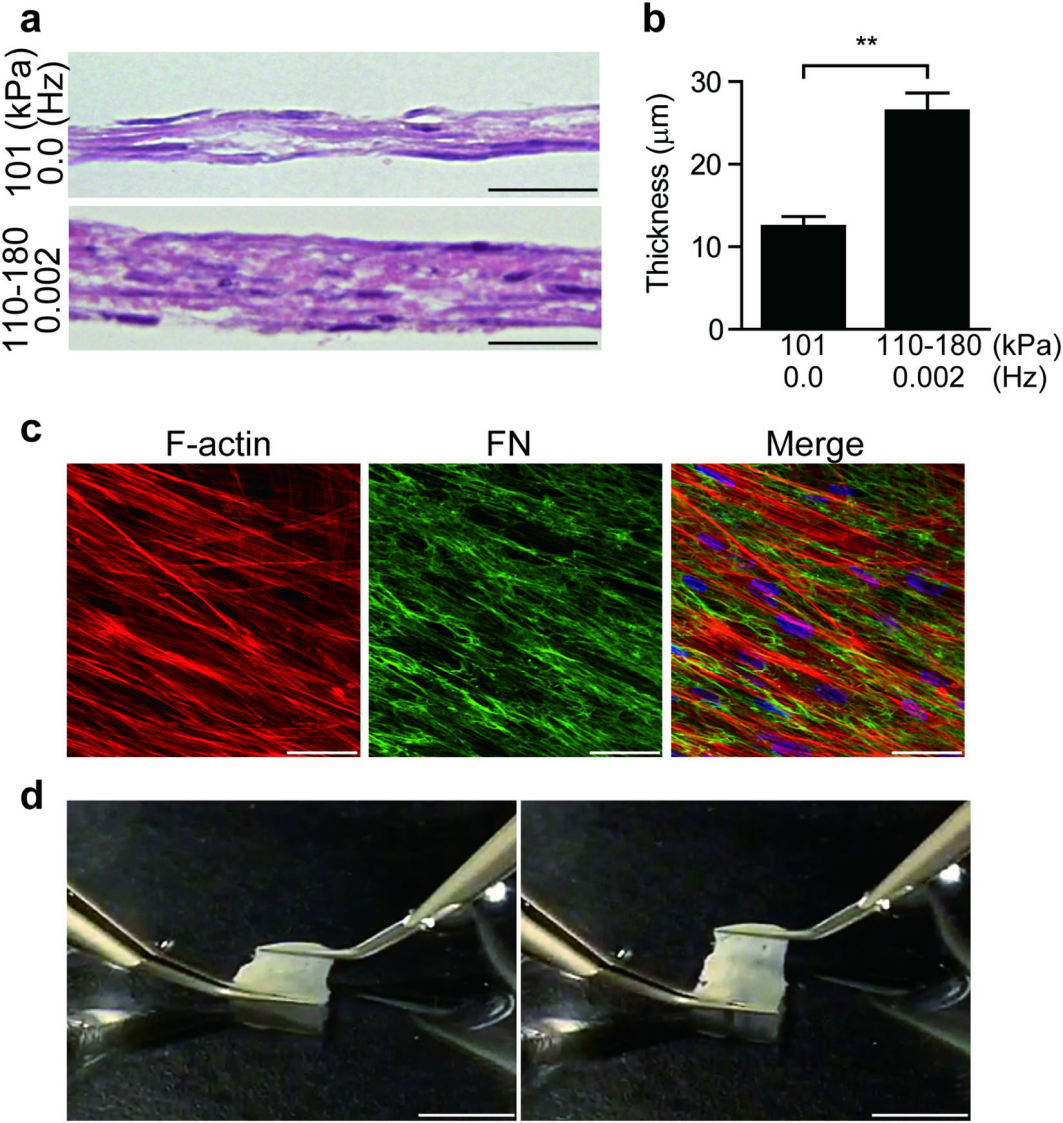
Layered structure of hUASMC constructs generated by repeated cell seeding and PHP. (**a**) Representative images of HE stain of hUASMC constructs. A lower panel shows a layered hUASMC construct generated by repeated cell seeding under PHP (110–180 kPa, 0.002 Hz) four times. The structure of hUASMCs made by the same time-course of repeated cell seeding, but without pressurization, is shown in an upper panel (101 kPa, 0.0 Hz). Scale bars: 20 μm. (**b**) Quantification of thickness of hUASMC constructs generated under 101 kPa, 0.0 Hz (atmospheric pressure) and PHP, respectively. n = 4, **p < 0.001. (**c**) Images of immunofluorescent stain for F-actin and FN in the layered structure of hUASMCs generated by PHP are shown. Scale bars: 50 μm. (**d**) Images of the layered structure of hUASMCs generated by PHP. Stretched and unstretched hUASMC constructs are shown in the right and left panels, respectively. Scale bars: 5 mm. Values are shown as the mean ± standard error of the mean (SEM) of independent experiments. Data were analyzed using two-tailed unpaired Student’s *t*-test.

**Figure 6:**
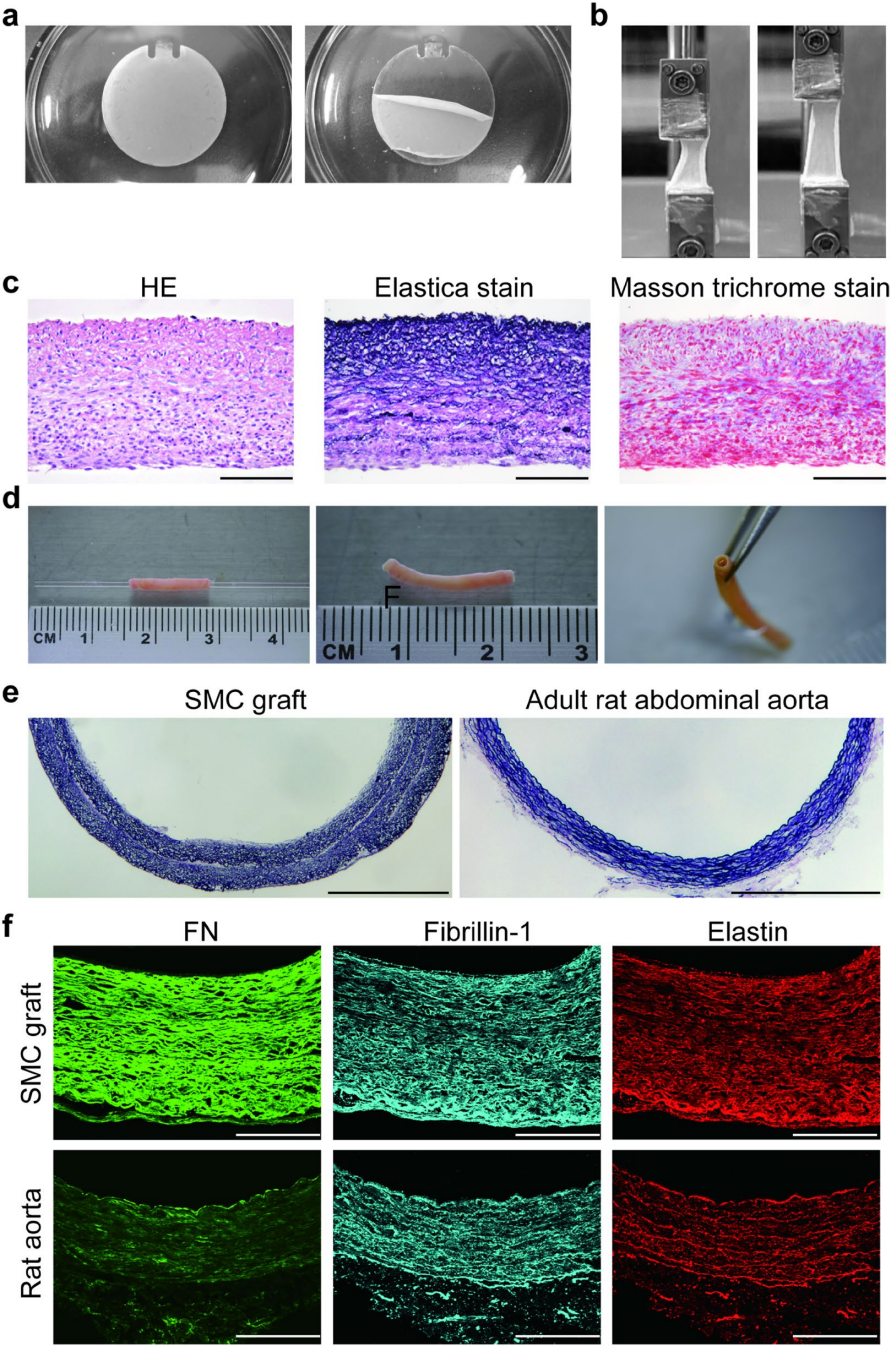
Fabrication of rat tubular medial graft by means of PHP. (**a**) Fifteen-layered rat aortic SMC constructs on a 30-mm disc. (**b**) Stretched and unstretched rat aortic SMC constructs. (**c**) Hematoxylin-eosin (HE), Elastica van Gieson (Elastica), and Masson trichrome stains of rat aortic SMC constructs. (**d**) Ten-layered structure of rat aortic SMC construct was wrapped around a glass tube (external diameter: 1 mm) (a left panel). Two weeks after the wrapped tubular medial graft was incubated with culture media containing ascorbic acid (40 μmol/L), the glass tube was removed (middle and right panels). (**e**) Representative elastic van Gieson stain images of a tubular medial graft and adult rat abdominal aorta. Scale bars: 500 μm. (**f**) Immunofluorescent stain images of FN, fibrilin-1, and elastin in a tubular medial graft and adult rat abdominal aorta. Scale bars: 100 μm.

Based on our results, we further investigated the possible roles of PHP in the fabrication of the vessel-like layered structure of vascular SMCs. As shown in Supplemental Fig. 3, cells were plated on a culture dish and allowed to undergo sedimentation and stable adhesion under 101 kPa. Twenty-four hours after cell seeding, cells were exposed to PHP for 24 h, and then cells for the second layer were seeded on top of the first layer. We repeated this procedure four times and obtained multi-layered hUASMCs; the same procedure undertaken at 101 kPa produced less than four layers (Fig. 5a). The thickness of the PHP-induced layered structure SMCs was significantly greater than that of SMCs cultured under 101 kPa (Fig. 5b). Immunofluorescent staining demonstrated abundant stress fiber formation and FN fibrillogenesis in multilayers of hUASMCs (Fig. 5c). This layered structure of hUASMCs was stretched to approximately 150% uniaxial strain (n = 5) (Fig. 6d, and Supplemental movie 1) whereas the control hUASMC layers made at 101 kPa were thinner and easily torn (Supplemental movie 2). These data suggested that PHP promoted multilayered assemblies of vascular SMCs *in vitro*, which may be partly due to FN fibrillogenesis. FN fibrillogenesis is the initial step of elastic fiber formation^10^, and PCR data showed PHP-induced expression of lysyl oxidase (Fig. 4) which is crosslinking enzyme of elastin and collagen. Elastin and collagen may also be involved in PHP-induced layered structure of SMCs.

### Construction of medial graft using rat aortic SMCs

To evaluate PHP-induced multilayer medial constructs *in vivo*, we performed repeated cell seeding and PHP according to the same protocol as hUASMCs using rat neonatal aortic SMCs, and obtained ten layers of medial constructs (Fig. 6a). Multi-layered rat vascular SMCs grown under periodic HP were stretched to approximately 180% under uniaxial strain (n = 4, Fig. 6b), and contained elastic fibers and collagens (Fig. 6c). These constructs were easily detached from the cell disk using a cell scraper and wrapped around the glass tube as shown in Fig. 6d. Since ascorbic acid induces abundant ECM, i.e., collagens^11^, the rolled medial constructs were then incubated with 10% fetal bovine serum (FBS)/Dulbecco’s modified Eagle’s medium (DMEM) supplemented with ascorbic acid for another 2 weeks under 101 kPa.

The middle and right panels of Fig. 6d show tubular medial constructs after glass tube removal. Elastica van Gieson staining revealed layered elastic fiber formation in this graft that is equivalent to that in adult rat abdominal aorta (Fig. 6e). Furthermore, the laminar pattern of FN, fibrillin-1 and elastin immunofluorescent signals were observed in the graft (Fig. 6f).

### Mechanical properties of rat tubular grafts

**Figure 7:**
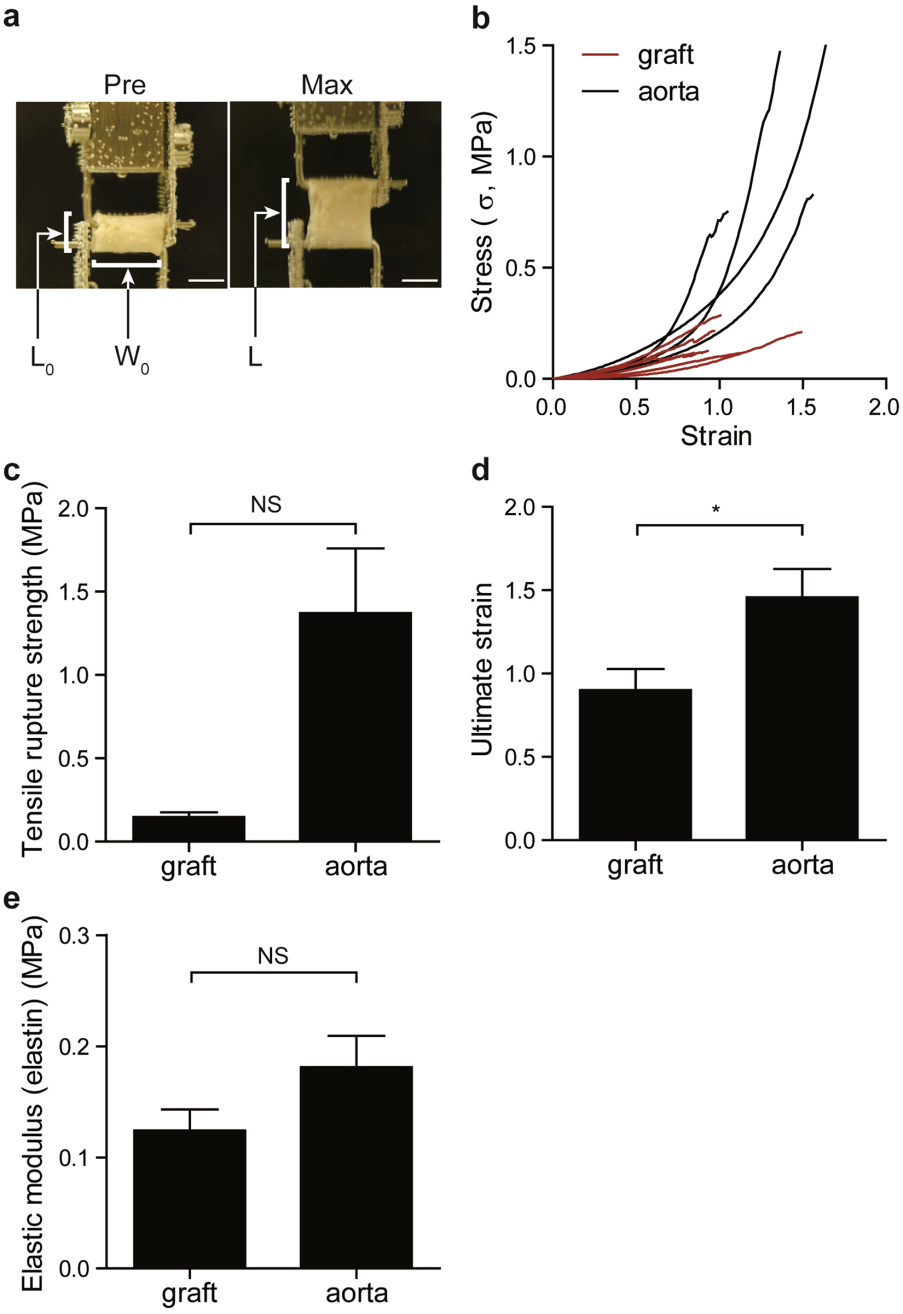
Mechanical properties of the tubular grafts. (**a**) Representative images of mechanical testing of tubular medial grafts. A tubular graft was placed around two parallel hooks made of stainless steel wire. A graft before pulling (Pre) and a maximum stretched tubular graft (Max). W_0_ and L_0_ indicate initial width of sample and sample length, respectively. L indicates sample length during the pulling. Scale bars: 3 mm. (**b**) Stress-strain behaviors of the tubular grafts and the tunica media of the adult rat thoracic aortae. (**c**–**e**) Comparison of tensile rupture strength, ultimate strain, and elastic modulus in elastin region between the tubular grafts and the aortae. Values are shown as the mean ± standard error of the mean (SEM) of independent experiments. Data were analyzed using two-tailed unpaired Student’s *t*-test with (**c**) or without (**d** and **e**) Welch’s correction. n = 4 and 8 (rat aorta and grafts, respectively); NS, not significant; *p < 0.05.

Ringlets of tubular grafts (length: 3.5 mm, inner diameter: 1.0 mm) were pulled under tension to rupture (Fig. 7a). Stress-strain curves from rat tubular grafts and the tunica media of adult rat thoracic aortae obtained by enzymatic removal of adventitia (length: 3.5 mm, inner diameter: 1.0 mm) are shown in Fig. 7b. Tensile rupture strength was 0.19 ± 0.02 MPa (minimum: 0.10 MPa, maximum: 0.31 MPa, n = 8), which is equivalent to 1451 ± 159 mmHg (minimum: 734 mmHg, maximum: 2287 mmHg), although tensile rupture strength and ultimate strain of tubular grafts were smaller than those of the native rat aortic tunica media (Fig. 7c,d).

It is recognized that the initial part of the stress-strain relationship shows a linear behavior and elastin contributes mainly to this part of the stress-strain curve, and that the final phase of the stress-strain relationship is due to collagen fibers^12, 13^. These two regions affected by elastin and collagens were observed in the stress-strain curves of the rat aortae (Fig. 7b). In the rat tubular grafts, the initial part or the elastin dependent phase of stress-strain curves were similar to those in the rat aortae. However, these similarities were not observed during the collagen-dependent phase of the stress-strain curve (Fig. 7b). Elastin-associated elastic moduli of the grafts and the aortae were calculated using each plot’s initial linear region spanning 0–0.6 strain, because all stress-strain data plots were linear (R^2^ ≥ 0.90) in the 0–0.6 strain region (Supplemental Table 1). The values of elastin-associated elastic moduli were similar between the rat tubular grafts and the rat aortae (Fig. 7e, Supplemental Table 1). These data suggests that the elastic property of the tubular grafts is similar to that of the native aortae, but the amount of functional collagens in the tubular grafts were suboptimal.

### Implantation of rat arterial grafts

**Figure 8:**
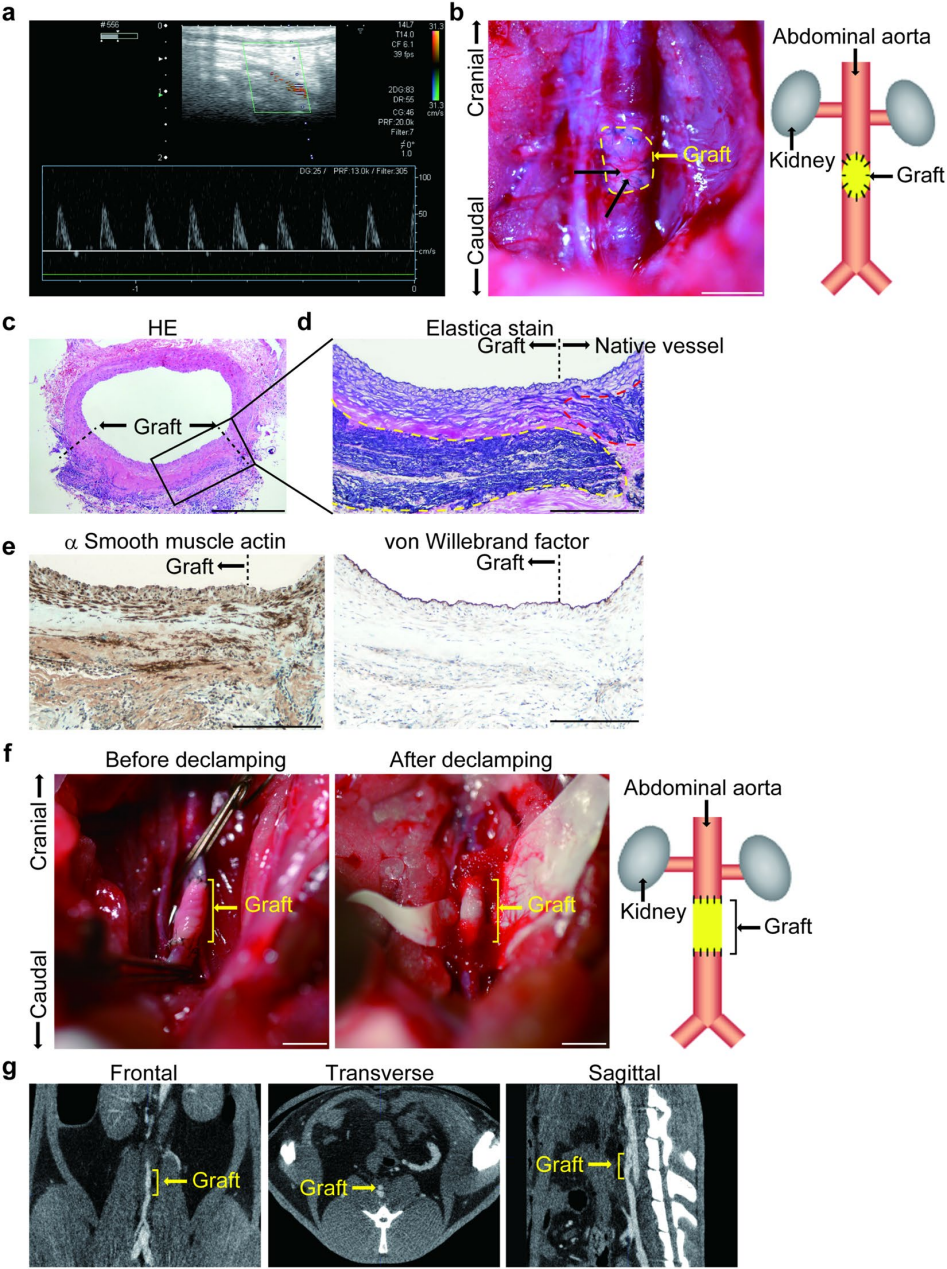
Implantation of medial grafts into rat abdominal aorta. (**a**) An oval shape of patch graft measuring 2.0 mm by 1.5 mm was sutured at an adult rat abdominal aorta in which the same size of aortic tissue was completely resected. Doppler echocardiography detected a non-stenotic flow pattern below the implantation site 2.5 months after implantation. (**b**) A patch graft implanted into the rat abdominal aorta is shown as a yellow dotted line. Arrowheads indicate capillary vessels on the surface of the patch graft. Scale bars: 2 mm. (**c**) An HE stain image of rat abdominal aorta implanted with a patch graft. Hitological data were obtained 2.5 month after implantation. The dotted lines indicate the area of the patch graft and the rest is native aortic tissue. Scale bar: 500 μm. (**d**) An elastica van Gieson stain image of the indicated area of (**c**). The black dotted line indicates a junction between a patch graft and native aortic tissue. The yellow and red black dotted lines indicate a patch graft and native aortic tissue, respectively. (**e**) Immunohistochemistry of α-smooth muscle actin and von Willebrand factor at the same area of (**d**). Scale bars of (**c**–**e**): 200 μm. (**f**) A tubular medial graft was implanted into a rat abdominal aorta. Scale bars: 2 mm. (**g**) Computed tomography (CT) scanning one day after surgery demonstrated the patency of the tubular medial graft.

For the first trial of graft implantation, we trimmed the aforementioned rat medial graft and obtained an oval-shaped patch graft measuring 2.0 mm by 1.5 mm. The patch graft was sutured at the adult rat abdominal aorta where the same size of aortic tissue including the intima, media, and adventitia was completely resected, as shown in Supplemental Fig. 4. Two-and-a-half months after implantation, Doppler echocardiography detected a non-stenotic flow pattern at the distal end of the implantation site (Fig. 8a). Patch grafts were successfully implanted (n = 3) and some capillary vessels were formed on the surface of the patch graft (Fig. 8b). Histological analysis was performed two-and-a-half months after implantation and revealed that no stenotic region was observed (Fig. 8c), and that the implanted patch graft expressed α-smooth muscle actin (αSMA) and contained abundant elastic fibers (Fig. 8d,e). The patch graft was covered with neointima (Fig. 8d,e). Luminal sides of the patch graft and the native aorta were continuously covered with endothelial cells as visualized by von Willebrand factor stain (Fig. 8e).

Lastly, we implanted a rat tubular graft into rat abdominal aorta by end-to-end anastomosis. The tubular grafts withstood arterial blood pressure (n = 4) (Fig. 8f). Computed tomography (CT) scanning demonstrated the patency of the medial graft one day after surgical operation (Fig. 8g). Two and half month after implantation, the tubular grafts were present but remodeled and exhibited aneurysmal formation (Supplemental Fig. 5). The grafts were obstructed with thrombus and systemic blood flow was maintained by collateral arteries. These data suggested that tubular grafts withstood at least one day and gradually dilated thereafter.

## Discussion

These data obtained from *in vitro* experiments demonstrated that PHP beyond the physiological condition promoted stress fiber formation and FN fibrillogenesis via the Rho/ROCK pathway, and the 3D assembly of SMCs in which expression levels of FN, fibrillin-1, fibrillin-2, fibulin-4, and lysyl oxidase mRNAs were increased. The combination of repeated cell seeding and supraphysiological PHP enabled the fabrication of SMC sheets composed of multiple layers *in vitro*. We adopted an approach using an autologous animal model for the *in vivo* evaluation of the SMC sheets. A patch graft of rat SMCs sutured into rat abdominal aorta was completely patent for at least 2.5 months, although the tubular graft implanted in rats as an abdominal interpositional graft withstood arterial blood pressure only in early acute phase.

Tunica media composed of a dense population of concentrically organized SMCs and ECMs largely determines arterial elasticity, and the decrease in arterial elastic properties is associated with cardiovascular risk factors^14^. In the case of permanent synthetic material graft implantation, compliance mismatch results in intimal hyperplasia of the grafts^15^. By some reports, up to 85% of PTFE graft failures result from intimal hyperplasia^16^. To develop biological grafts, most research utilized biodegradable synthetic, gel-based, or decellularised scaffold combined with vascular and non-vascular SMCs, and pericytes^17–25^. However, biological media mimetics containing functional elastin that is equivalent to native arteries has not been developed. In this study, we performed ringlet tensile testing to define mechanical properties, and found that elastin-associated elastic modulus of rat tubular grafts was similar to that of tunica media of adult rat aortae. These results were supported by the data that supraphysiological PHP induced elastic fiber-related genes.

The average tensile rupture strength of tubular medial grafts was 1451 ± 159 mmHg, which did not reach that of the tunica media of rat aortae. Few studies have investigated the mechanical properties of tunica media of human small diameter vessels. It has been demonstrated that, in human coronary arteries, the tensile stress of tunica media was threefold smaller than the ultimate tensile stresses in the adventitia^26^. Since the burst pressure of internal mammary arteries was reported to be 3200 mmHg on average (534–5688 mmHg)^27^, the burst pressure of the tunica media is estimated to be 178 to 1896 mmHg. The burst pressure of human saphenous vein containing all three layers was reported to be 1599 ± 877 mmHg^27^. Although the mechanical strength of these human vessels is similar to the burst pressure range of the tubular grafts, our data on tensile rupture strength may be overestimated because it was suggested to have a moderate tendency to overestimate burst pressure compared to the direct measurement of longer tubular grafts^27^. Data of stress-strain curves from the rat tubular grafts suggested that amount of functional collagen was much smaller in the grafts than in the rat aortae. As expected, tubular grafts were unable to withstand arterial pressures for longer periods *in vivo*. Elastic properties in the tube grafts appeared to be satisfactory, but increasing tensile strength is required for future clinical use. Since we confirmed in preliminary study that PHP can fabricate a layered structure of fibroblasts and mesenchymal cells, PHP-mediated fabrication of adventitia using these cells may have clinical applications. Our settings of PHP did not increase collagen expression in SMCs. Suitable settings of PHP for collagen should also be investigated in the future.

Vessels are continuously exposed to hemodynamic forces, such as shear stress, cyclic strain, and hydrostatic pressure. Therefore, the potentials of these mechanical stresses to enhance the fabrication of artificial vascular grafts have been widely studied^11, 20, 23, 28–30^. Fibroblast grafts (inner diameter: 4.2 mm) generated by sheet-based tissue engineering were exposed to pulsatile flow that increased from 3 ml/min to 150 ml/min for 3 days and were successfully implanted into animals and humans^11, 28^. Pulsed flow-stretch bioreactors were applied to scaffold-based tissue-engineered vascular graft (TEVG), in which 5 to 10% distension was used^20, 23, 29, 30^. In addition to the effect of fluid flow, a recent study investigated the effect of hydrostatic pressure^6^. Tubular vascular grafts composed of poly (glycerol sebacate) (PGS) scaffold and primary adult baboon arterial SMCs were cultured at 10 mmHg or 120 mmHg with the same fluid flow rate, and it was found that the hydrostatic pressure significantly increased the construct burst pressure, collagen, and insoluble elastin content in TEBVs^6^. Although these results suggest that the physiological range of hemodynamic forces provide support for the improvement of arterial graft mechanical properties, these physiological mechanical stresses *per se* did not assemble vascular cells into constructs.

In the current study, we attempted to apply an unprecedented range of hydrostatic pressure in the cardiovascular field on vascular SMCs for arterial construct fabrication. We found that 110 to 180 kPa (equivalent to 65–590 mmHg) PHP significantly increased stress fiber formation and FN fibrillogenesis, and the expression of multiple elastic fiber-related genes in hUASMCs. A previous study demonstrated that increasing the pulsatile hydrostatic pressure to the pathological range of magnitude at a physiologic frequency increased the ECM content in aortic valve leaflets^5^. The authors investigated up to 150 to 190 mmHg and found the maximum response in this setting, suggesting that a higher magnitude of the PHP setting may further increase the ECM content. No studies have examined the hydrostatic pressure beyond the pathological setting (i.e., hypertension) in vascular SMCs, but the burst pressure of the native internal mammary artery is more than 3000 mmHg^27^ and there is a wide gap between the previously tested PHP range and burst pressure. The PHP setting in this study was over the pathophysiological range and appears to have the potential to regulate the ECM complex.

In contrast to the PHP amplitude setting, we applied lower frequency than the physiological range, because our system allowed the frequency to be less than 0.25 Hz (equivalent to 15 beats per min). Interestingly, our findings suggested that the lowest frequency (0.002 Hz) induced a preferable effect on ECM fiber formation in monolayer vascular SMCs and 3D-SMC assembly. The previous study using aortic valve leaflets investigated the effect of PHP frequency^5^. Collagen and glycoaminoclycan contents were increased at 0.5 Hz, but not 2 Hz^5^. These data support our findings that lower frequency enhanced ECM regulation.

The effect of PHP frequency on cell proliferation was reported to be opposite to that for ECM production^5^. Excessive proliferation may cause intimal hyperplasia and is not beneficial for artificial vascular grafts. It has been reported that the physiological frequency of PHP and constant hydrostatic pressure induced bovine and human aortic SMC proliferation, respectively^31–33^. However, one study investigating the frequency-dependent effect demonstrated that DNA synthesis was significantly increased in higher frequency PHP (2 Hz), but not at lower frequency (0.5 Hz)^27^. Although we did not examine DNA synthesis, the much lower frequency setting in our study may avoid excessive proliferation.

The molecular mechanisms of hydrostatic pressure-induced phenotypic changes in vascular SMCs, such as ECM production, are not fully understood, although emerging evidence has shown the preferable effects of mechanical stress on arterial graft fabrication. Here, we demonstrated that PHP induced actin stress fiber formation and FN fibrillogenesis via Rho/ROCK pathways. Hydrostatic pressure-induced stress fiber formation has been reported in other cell types, including bone marrow mesenchymal stem cells^9, 34^, synovial fibroblasts^35^, and aortic endothelial cells^36^. Activated RhoA, a ras-related GTP-binding protein, is recognized to stimulate the appearance of stress fibers through the enhancement of cell contractility^37, 38^. Indeed, hydrostatic pressurization induced Rho activation in vascular SMCs^39^, and a study on bone marrow mesenchymal stem cells demonstrated that hydrostatic pressure-induced actin stress fiber formation was mediated by the RhoA pathway^9^. Rho-stimulating bundles of actin filaments and contractility expose self-assembly sites within FN, resulting in FN fibrillogenesis, which is an orchestrator of fibrillin-1 microfibril assembly^40^ and collagen I fiber formation^41^. The interaction of FN and fibrillin-1 is a requisite process for elastic fiber formation^10^. Although few studies have investigated the effect of hydrostatic pressure on FN fibrillogenesis in vascular cells, one paper reported that hydrostatic pressure promoted FN fibril formation and FN protein expression in bovine aortic ECs^36^, which supports our findings.

In this study, PHP induced layered arterial media constructs consisting of hUASMCs and rat neonatal aortic SMCs *in vitro* without the need for synthetic scaffolds or exogenous materials, because neonatal cells have much higher expression of elastic fiber-related genes^3^. Human UASMCs possessed ECM secretion ability and may be a potential source of TEBVs when HLA typing matches a donor and recipient. Alternative cell sources must be identified, however, to generate clinically relevant tissues due to problems related to self-renewal capacity and the collection of a large number of umbilical cords for HLA matching. Recent studies have demonstrated the utilization of adult stem cells^42–44^ and induced-pluripotent stem cells^45^ for TEBV. It has been reported that hydrostatic pressurization promoted stress fiber formation in bone marrow mesenchymal stem cells^9, 34^. Based on these findings, hydrostatic pressure-mediated tissue development characterized by the sequential deposition of multiple ECM proteins into an ordered structure of interwoven fibrils may be applicable for stem cells. In future research, cells with a high self-renewal capacity (i.e., adult stem cells and induced-pluripotent stem cells) would be utilized in this PHP system. In addition, fabrication of endothelial cell-covered medial constructs will be required. Although our relatively small grafts were completely covered with endothelial cells, larger medial constructs for clinical use may not be fully covered with endothelial cells. Our previous study demonstrated that endothelial cells were adhered on the top of 3D-multilayered vascular SMC constructs when FN fibrillogenesis was formed on SMCs^4^. Based on these findings, it may be feasible that endothelial cells are layered on SMC multilayers by PHP. The further study of fabrication of endothelial cell-covered medial constructs needs to be performed.

## Methods

### Reagents

Anti-FN rabbit polyclonal antibody was purchased from Abcam (Cambridge, UK); Anti-F-actin rhodamine conjugated antibody, Hoechst 33342 solution, Alexa Fluor 488 goat anti-rabbit IgG, Alexa Fluor 594 goat anti-rabbit IgG, and Alexa Fluor 488 donkey anti-goat IgG were purchased from Thermo Fisher Scientific (Waltham, MA); Anti-elastin antibody (RA75) was purchased from Elastin Products Co. Inc. (Owensville, MI); Anti-α SMA (A2547) were purchased from Sigma (St. Louis, MO); Anti-von Willebrand factor antibody was purchased from Dako Cytomation (Glostrup, Denmark); Anti-fibrillin-1 was kindly provided by Professor T. Nakamura (Kansai Medical University, Japan); Y27632 and C3 were purchased from Cayman Chemical (Ann Arbor, MI) and Cytoskeleton, Inc. (Denver, CO), respectively.

### Cell culture

Human umbilical artery SMCs (hUASMCs) were obtained from Lonza (Walkersville, MD). Vascular SMCs in primary culture were obtained from the aorta of rat neonates (day 0) as previously described^46, 47^. Briefly, the minced tissues were digested with a collagenase-dispase enzyme mixture at 37 °C for 20 min. The cell suspensions were then centrifuged, and the medium was changed to a collagenase II enzyme mixture. After 12 min of incubation at 37 °C, cell suspensions were plated on 35 mm poly-L-lysine-coated dishes. The growth medium for both hUASMCs and rat aortic SMCs contained Dulbecco’s modified Eagle’s medium (DMEM) with 10% fetal bovine serum (FBS), 100 U/ml penicillin, and 100 mg/ml streptomycin (Invitrogen, Carlsbad, CA). The confluent SMCs were used at passages 6–12. All cells were cultured in a moist tissue culture incubator at 37 °C in 5% CO_2_-95% atmospheric mixed air until exposure to PHP.

### Animals

Neonate and adult Wistar rats were obtained from Japan SLC, Inc. (Shizuoka, Japan). All animal studies including cell isolation and graft implantation were approved by the Animal Care and Use Committee of Yokohama City University (reference number: F-A-16-009). All animals were cared for in compliance with the guiding principles of the America Physiological Society.

### Periodic hydrostatic pressurization system

Figure 1 shows an overview of the experimental system, which is mainly composed of a pressure vessel containing a cell culture dish, a compressor for supplying the pressurized air to the pressure vessel, a controller for regulating the flow of pressurized air to the pressure vessel, and a PC for controlling the frequency and the amplitude of pressure. The compressor was supplied with moist air kept at 37 °C and appropriate CO_2_ concentration. The compressed air controller can change the frequency from zero up to 0.25 Hz and the amplitude can be changed from zero to 1 MPa by adjusting the pressure in a regulator tank. Since pressurization increases the dissolved CO_2_ concentration in culture media, we changed the CO_2_ concentration from 3.6% to 5% in supplied air until the pH levels in cell culture media reached 7.4. Levels of pH in cell culture media were measured using an i-STAT blood analysis system (Abbott Labs, Princeton, NJ). A pressure vessel was also kept at 37 °C.

### Immunocytochemistry

hUASMCs were seeded on 12 mm uncoated glass coverslips at a density of 15000 cells per coverslip and cultured with 10% FBS/DMEM under AP. Twenty-four hours after seeding, the cells were exposed to various settings of PHP or maintained under AP for 24 h. Cells were then fixed in 10% buffered formalin for 10 min and subjected to immunocytochemistry. Immunocytochemical analysis was performed as previously described^48^. Briefly, fixed cells were washed twice with PBS, and permeabilized in 0.3% Triton X-100 for 10 min. Cells were then washed twice with Tween 20 (0.1%)/PBS and incubated with 1% BSA/Tween 20 (0.1%)/PBS for 20 min, and incubated with anti-FN and rhodamine-conjugated anti-F-actin antibodies for 24 h at 4 °C. After three washes with Tween 20/PBS, cells were incubated with a secondary antibody, Alexa Fluor 488 anti-rabbit IgG, for 1 h. After six washes with Tween 20/PBS, DNA was stained with Hoechst 33342 solution. After two washes with PBS, coverslips were mounted for microscopic imaging. Images were obtained with a scanning laser microscope (Olympus FV1000) and software Olympus FluoView (Tokyo, Japan). Stress-fiber polymerization and fibronectin fibrillogenesis were evaluated by measuring the intensity of fluorescent signals. Quantitative analysis of fluorescent intensity was performed using Olympus FluoView software.

### Gene expression analysis

hUASMC suspension in 10% FBS/DMEM was transferred to uncoated container at a density of 1900 cells/mm^2^. The cells were able to access compressed or atmospheric air through top part of the container. Varying PHP was applied at 0.25, 0.05, 0.01, and 0.002 Hz with a 110 to 130 kPa offset and a 110 to 500 kPa maximum for 18 h. hUASMCs were cultured under 101 kPa for 18 h as controls.

Isolation of total RNA, generation of cDNA, and RT-PCR analysis were carried out as described previously^46^. The primers were designed based on human nucleotide sequences of fibronectin (FN), tropoelastin, fibrillin-1, fibrillin-2, fibulin-4, fibulin-5, lysyl oxidase, collagen type I α1, collagen type III α1, and collagen type IV α1. Sequences for the primers are listed in Supplemental Table 2. Each primer set was designed between multiple exons, and PCR products were confirmed by sequencing. The abundance of each gene was determined relative to the 18S transcript.

### Fabrication of layered structure of arterial construct

A cell disk LF (Sumitomo Bakelite, Tochigi, Japan) was coated with FN (0.2 mg/ml) for 30 min at 37 °C. To fabricate the first layer of SMCs, cells were seeded on a 30 mm cell culture disk at a density of 1 × 10^6^ cells per dish (1415 cells/mm^2^) and cultured with 10% FBS/DMEM under 101 kPa. Twenty-four hours after seeding, the cells were exposed to PHP (110–180 kPa, 0.002 Hz) for 24 h. To fabricate the second layer, the same procedure as the first layer (i.e., cell seeding following 24 h culture under 101 kPa and subsequent 24 h exposure to PHP) was performed. This seeding and pressurization was repeated until the intended number of layers of vascular SMC structure was obtained.

### Fabrication of tubular medial graft

First, the layered structures of rat aortic SMCs were obtained by the abovementioned method. Primary culture of rat aortic SMCs (passage: 6–12) were seeded on a 30 mm FN-coated cell culture disk at a density of 1 × 10^6^ cells per dish (1415 cells/mm^2^) and cultured with 10% FBS/DMEM under 101 kPa. Twenty-four hours after seeding, the cells were exposed to PHP (110–180 kPa, 0.002 Hz) for 24 h. To fabricate the second layer, the same procedure as the first layer was performed. This seeding and pressurization was repeated for ten times to obtain ten layers of medial vascular SMC sheet.

The medial vascular SMC sheet was detached from cell disks and wrapped around glass tube (external diameter: 1 mm). Two weeks after the wrapped SMC construct was incubated with culture media (10% FBS/DMEM) containing 2-*O*-α-D-glucopyranosyl-L-ascorbic acid (40 μmol/L, Tokyo Chemical Industry Co., Ltd., Tokyo, Japan) under 101 kPa, the glass tube was removed. Total 34 days including 20 days for fabrication of ten layer SMC sheet and additional 14 days for graft maturation were taken for graft fabrication.

### Histological analysis

Paraffin-embedded blocks containing the layered structure of hUASMC constructs, rat tubular medial grafts, and rat abdominal aortic tissues were cut into 4-μm-thick sections and placed on glass slides. For immunohistochemistry, the specimens were de-paraffinized, rehydrated, and incubated with primary antibodies against αSMA or von Willebrand factor at 4 °C overnight. After they were washed with 0.1 M PBS, the slides were incubated for 30 min in biotinylated rabbit Ab (Vectastain Elite ABC IgG kit, Vector Labs, Burlingame, CA, USA). The targeted proteins were demonstrated with DAB (Dako, Glostrup, Denmark). The slides were counterstained with Mayer’s hematoxylin. Negative stain was confirmed by the omission of primary antibodies. For immunofluorescent stain, de-paraffinized slides were incubated with primary antibodies against FN, fibrilin-1, or elastin at 4 °C overnight. After three washes with Tween 20/PBS, specimens were incubated with secondary antibodies, Alexa Fluor 488 goat anti-rabbit IgG, Alexa Fluor 594 goat anti-rabbit IgG, or Alexa Fluor 488 donkey anti-goat IgG for 1 h. After six washes with Tween 20/PBS, DNA was stained with Hoechst 33342 solution. After two washes with PBS, coverslips were mounted for microscopic imaging. Images were obtained using a scanning laser microscope (Olympus FV1000) and Olympus FluoView software (Tokyo, Japan). Hematoxylin eosin (HE) stain and elastica van Geison stain were performed for morphological analysis and evaluation of elastic fiber formation as described previously^49^.

### Mechanical properties

The tensile strength of tubular medial grafts and the tunica media of adult rat thoracic aorta was measured using a DMT560 tissue puller (Danish MyoTechnology, Aarhus N, Denmark). Tunica media of rat aorta was obtained by enzymatic removal of the adventitia. The rats were euthanized with 100 mg/kg of pentobarbital purchased from Kyoritsu Seiyaku (Tokyo, Japan). The thoracic aortae were excised and the fat and surrounding connective tissue were removed. To obtain the tunica media, the aortae were digested by a collagenase enzyme mixture (1.5 mg/mL collagenase-dispase, 0.5 mg/mL elastase type II-A, 1 mg/mL trypsin inhibitor type I-S, and 1 mg/mL collagenase II) (Sigma-Aldrich, St. Louis, MO) at 37 °C for 3 min. The tunica media was separated from the adventitia using forceps. A 3.5-mm-long segment of each graft and the tunica media of adult rat aorta was placed around two parallel hooks made of stainless steel wire. The grafts and the rat aortae were pulled until complete rupture at 100 μm/s, and force and displacement were measured during the pulling by force and displacement sensors, respectively.

Stress is defined by the force per unit cross-sectional area of the sample, and was actually calculated by dividing the recorded force by the cross-sectional area of the sample (A_wall_) using the initial geometric dimension of the sample. To measure the initial wall thickness (h_0_) and length (L_0_) of samples, adjacent samples were cut in paraffin blocks and imaged under a microscope. The initial width of the samples (W_0_) was measured in the first video frame (σ = 0, or zero-stress state). The initial sample volume was obtained as follows (1):

1 $${V}_{0}=2{h}_{0}\ast {W}_{0}\ast \,{L}_{0}$$

Because volume of samples (V) was constant during the pulling, cross-sectional area during the pulling (A_wall_) was obtained as follows (2):

2 $${A}_{wall}=2h\ast W=\frac{{V}_{0}}{L}$$

where h and W are wall thickness and width of samples during the pulling, respectively. Sample length during the pulling (L) was measured by the position sensor of a DMT560 tissue puller.

The peak force was the greatest force attained during pulling. Tensile rupture strength is defined as peak force/tissue cross-sectional area. Ultimate strain is defined as strain value at peak force. Elastin-associated elastic moduli of the grafts and the aortae were calculated using each plot’s initial linear region spanning 0–0.6 strain, because all stress-strain data plots were linear (R^2^ ≥ 0.90) in the 0–0.6 strain region (Supplemental Table 1).

### Implantation

Male Wistar rats (320–360 g body weight) were anesthetized with intraperitoneal injection of tribromoethanol (250 mg/kg). A midline laparotomy incision was made and the abdominal aorta exposed below the renal arteries. Microclamps were applied to the infrarenal aorta, proximally and distally. A patch graft trimmed to 2.0 mm × 1.5 mm was sutured at the adult rat abdominal aorta in which the same size of all three layers of aortic vessel wall, such as tunica intima, tunica media, and tunica adventitia, was completely resected. For implantation of tubular medial construct, microclamps were applied to the infrarenal aorta, and the vessel was sectioned between the clamps. A tubular graft trimmed to 6 to 7 mm long was implanted at the gap of the aorta and then sutured in an end-to-end interrupted anastomotic pattern with 10–0 nylon monofilament suture (Muranaka Medical Instruments Co., Ltd., Osaka, Japan). An average of 10 to 12 stitches per anastomosis was utilized. After the patch graft or tubular medial graft was anastomosed, the clamps were released and patency was verified by direct observation. Finally, the muscle layer and skin were closed with 5–0 nylon monofilament suture (Akiyama Medical Mfg. Co., Ltd., Tokyo, Japan). Mechanical failure was considered when obvious dilation could be observed at the time of declamping.

### Echocardiography

Rats were anesthetized with tribromoethanol (250 mg/kg) intraperitoneal injection. Echocardiography was performed with ultrasonography (Diagnostic Ultrasound System, Model SSA-700A, Toshiba, Tokyo, Japan). We measured pulsed-Doppler blood velocity across the junction between the distal end of a patch or tubular graft and the native rat abdominal aorta.

### Computed tomography

The rats were anesthetized by intraperitoneal injection of tribromoethanol (250 mg/kg). Five minutes after a single intravenous injection of 400 μL of a nanoparticulate contrast agent (ExiTron nano 12000; Miltenyi Biotec GmbH, Bergisch Gladbach, Germany), the rat was held in the cradle. The abdomen filled the field of view. The tube parameters were set to 90 kV and 160 mA (focal spot size 5 µm). The scanning protocol was programmed to acquire a total of 1200 projections per scan while continuously rotating the rat by 360°.

### Statistical analysis

All values are shown as the mean ± standard error of the mean (SEM) of independent experiments. Values were compared with a two-tailed Student’s *t*-test with or without Welch’s correction, and multiple-group comparisons were made using analysis of variance (ANOVA) followed by Dunnett’s multiple comparison test. A value of *p* < 0.05 was considered significant.

## Electronic supplementary material

Supplemental movie 1

Supplemental movie 2

Supplementary information
