# Supplementary material for: Arterial graft with elastic layer structure grown from cells
Source: Sci Rep. 2017 Mar 10;7:140. doi: 10.1038/s41598-017-00237-1 (PMC5428065; doi:10.1038/s41598-017-00237-1)

Supplementary Information

Arterial graft with elastic layer structure grown from cells

Utako Yokoyama, Yuta Tonooka, Ryoma Koretake, Taisuke Akimoto, Yuki Gonda, Junichi Saito, Masanari Umemura, Takayuki Fujita, Shinya Sakuma, Fumihito Arai, Makoto Kaneko, and Yoshihiro Ishikawa

Supplemental Figure legends

Supplemental Figure 1. The periodic hydrodynamic pressurization system

The experimental system was composed of a pressure vessel containing a cell culture dish, a compressor for supplying the pressurized air to the pressure vessel, a controller for regulating the flow of pressurized air to the pressure vessel, and a PC for controlling the frequency and the amplitude of pressure. A compressor was supplied with moist air kept at 37°C and appropriate CO_2 concentration. To adjust the pH to 7.4 in the cell culture medium, we changed the CO_2 concentration from 3.6% to 5% in supplied air. A pressure vessel was also kept at 37°C.

Supplemental Figure 2. PHP-induced gene expression

(**a-e**) Suspensions of hUASMCs were exposed to pressure from 130 kPa to 500 kPa in which lower pressure was kept at 110 kPa and frequency was maintained at 0.002 Hz. Expression levels of tropoelastin, fibulin-5, collagen type I α1, collagen type III α1, and collagen type IV α1 mRNAs were quantified by real-time RT-PCR. (**f-j**) Suspensions of hUASMCs were exposed to pressure from 0.25 Hz to 0.002 Hz in which lower and higher

pressure was kept at 110 kPa and 180 kPa, respectively. Genes were quantified in the same way as (a-e). n = 4-7, NS, not significant vs. 101 kPa, 0.0 Hz (= atmospheric pressure). Values are shown as the mean ± standard error of the mean (SEM) of independent experiments. Data were analyzed using analysis of variance (ANOVA) followed by Dunnett's multiple comparison test. No significant difference was detected in all genes.

Supplemental Figure 3. Procedure of fabrication of layered structure of vascular SMCs

Vascular SMCs were plated on a culture dish and allowed to undergo sedimentation and stable adhesion under 101 kPa, 0.0 Hz (= atmospheric pressure) for 24 h, followed by PHP exposure for 24 h. To fabricate the second layer, cells for the second layer were seeded on the top of the first layer. This procedure was repeated to obtain the intended number of layers in the layered SMC structure.

Supplemental Figure 4. Procedure of the patch graft implantation

(**a**) An oval shape of all three layers of aortic vessel wall including tunica intima, tunica

media, and tunica adventitia, measuring 2.0 mm by 1.5 mm was resected before implantation. (**b**) An oval shape of patch graft measuring 2.0 mm by 1.5 mm was sutured at an adult rat abdominal aorta. Yellow dotted line indicates the patch graft.

Supplemental Figure 5. Histological analysis of implanted tubular graft

(**a**) A representative elastica van Gieson stain image of middle portion of the tubular grafts 2.5 month after implantation. Elastic fibers of implanted tubular graft were visible in dark purple. Internal lumen was occluded with thrombus. Scale bar: 500 μm.

Supplemental Movie legends

Supplementary movie 1. A multilayered hUASMC sheet fabricated by HP (110-180 kPa, 0.002Hz) was stretched to approximately 150% under uniaxial strain.

Supplementary movie 2. A layered hUASMC sheet fabricated under 101 kPa, 0.0 Hz (= atmospheric pressure) was easily torn.

Supplemental Table 1. Elastic modules of each graft.

	Elastic modules		R^2
	Slope	95% Confidence intervals	
graft 1	0.036	0.034 - 0.039	0.935
graft 2	0.163	0.154 - 0.172	0.957
graft 3	0.203	0.190 - 0.215	0.954
graft 4	0.145	0.140 - 0.151	0.984
graft 5	0.110	0.105 - 0.115	0.947
graft 6	0.131	0.127 - 0.134	0.989
graft 7	0.063	0.060 - 0.066	0.940
graft 8	0.142	0.141 - 0.144	0.997
aorta 1	0.186	0.170 - 0.201	0.918
aorta 2	0.128	0.122 - 0.133	0.974
aorta 3	0.154	0.146 - 0.162	0.968
aorta 4	0.258	0.247 - 0.269	0.979

Supplemental Table 2. Oligonucleotides used for RT-PCR.

mRNA description	Accession number	Forward (5'-3')<br>Reverse (5'-3')	Size (bp)
fibronectin	NM_002026	GAGAATAAGCTGTACCATCGCAA<br>CGACCACATAGGAAGTCCCAG	200
tropoelastin	NM_000501	AAGACCTGGCTTCGGATTG<br>CTCTTGTTTCCTTGCCCTG	244
fibrillin-1	NM_000138	CCTGGCTACCATTCAACTCC<br>ATAACACAAGCACCTGTACTCTCC	249
fibrillin-2	NM_001999	TGAATGGTGGGACCTGTG<br>CTGGTTGTTGACCTGAGTGAA	209
fibulin-4	NM_016938	CATAGACGAGTGCCGCTACC<br>AGGAAGGTCCCATAGGAGTTGA	183
fibulin-5	NM_006329	TTCCCGCTGACATCTTCC<br>GCTGCCTCTGAAGTTGATGAC	221
lysyl oxidase	NM_001178102	CAGTACAGCATACAGGGCAGA<br>TGGCATCAAGCAGGTCATAG	191
collagen type I α1	NM_000088	AGAGGAAGGAAAGCGAGGA<br>ACCAGGCAGACCAGCTTCAC	217
collagen type III α1	NM_000090	CTGAAGGGCAGGGAACAAC<br>TAGGACTGACCAAGATGGGAAC	207
collagen type IV α1	NM_001845	CCAGGGGTCGGAGAGAAAG<br>GGTCCTGTGCCTATAACAATTCC	203

Supplemental Figure 1

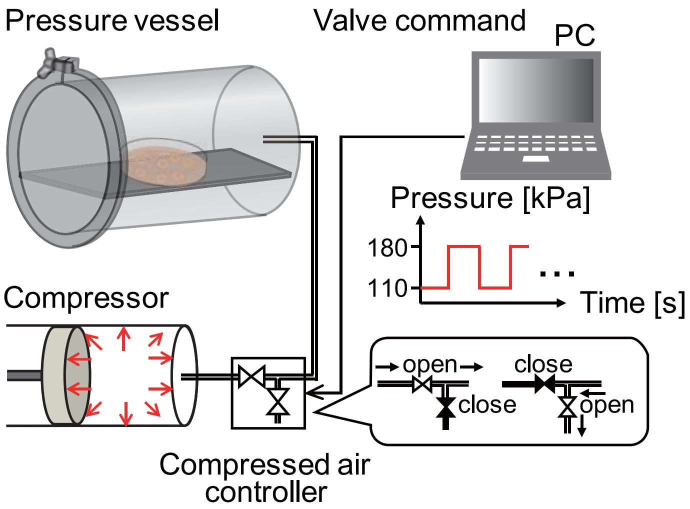

Supplimental Figure 3

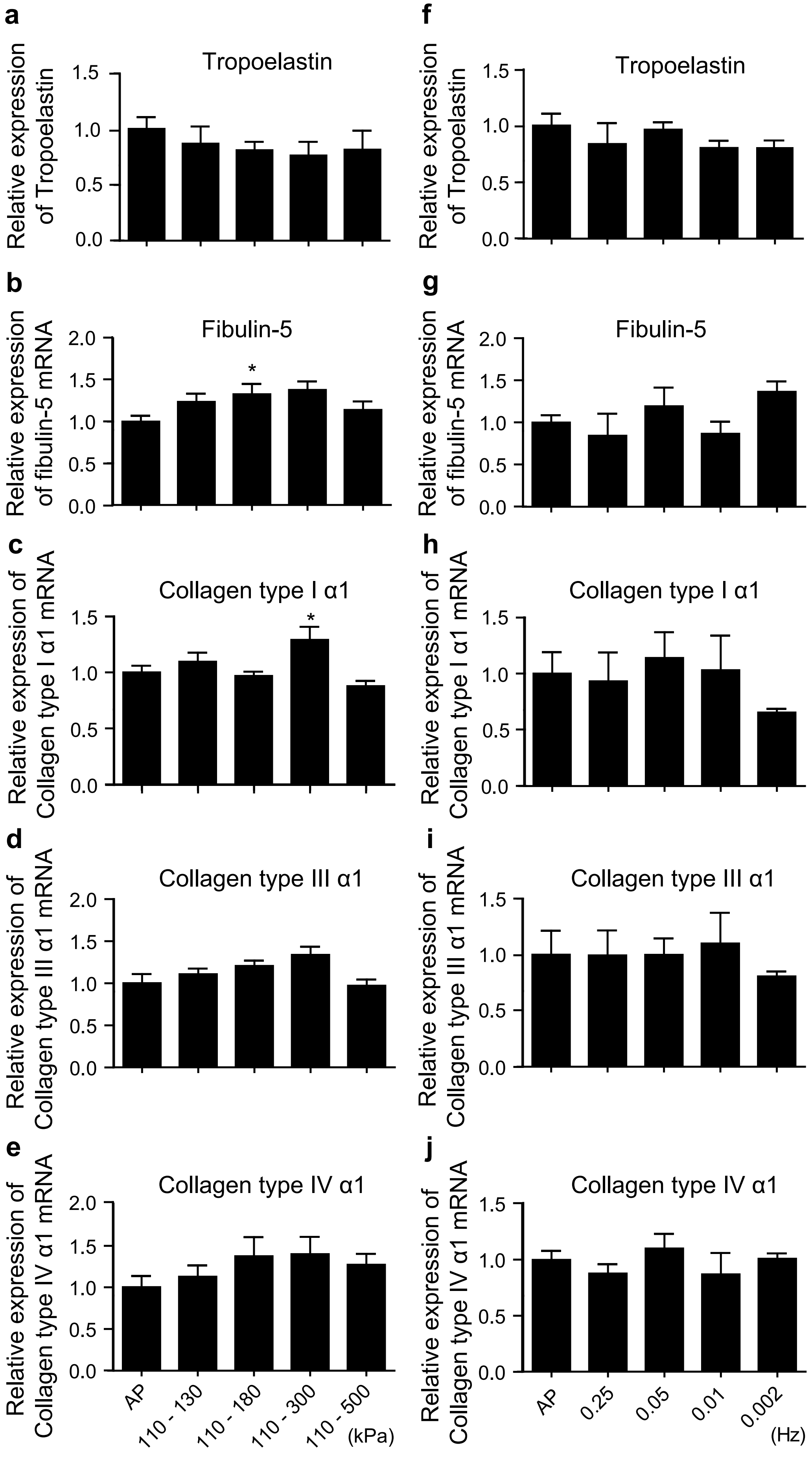

Supplemental Figure 3

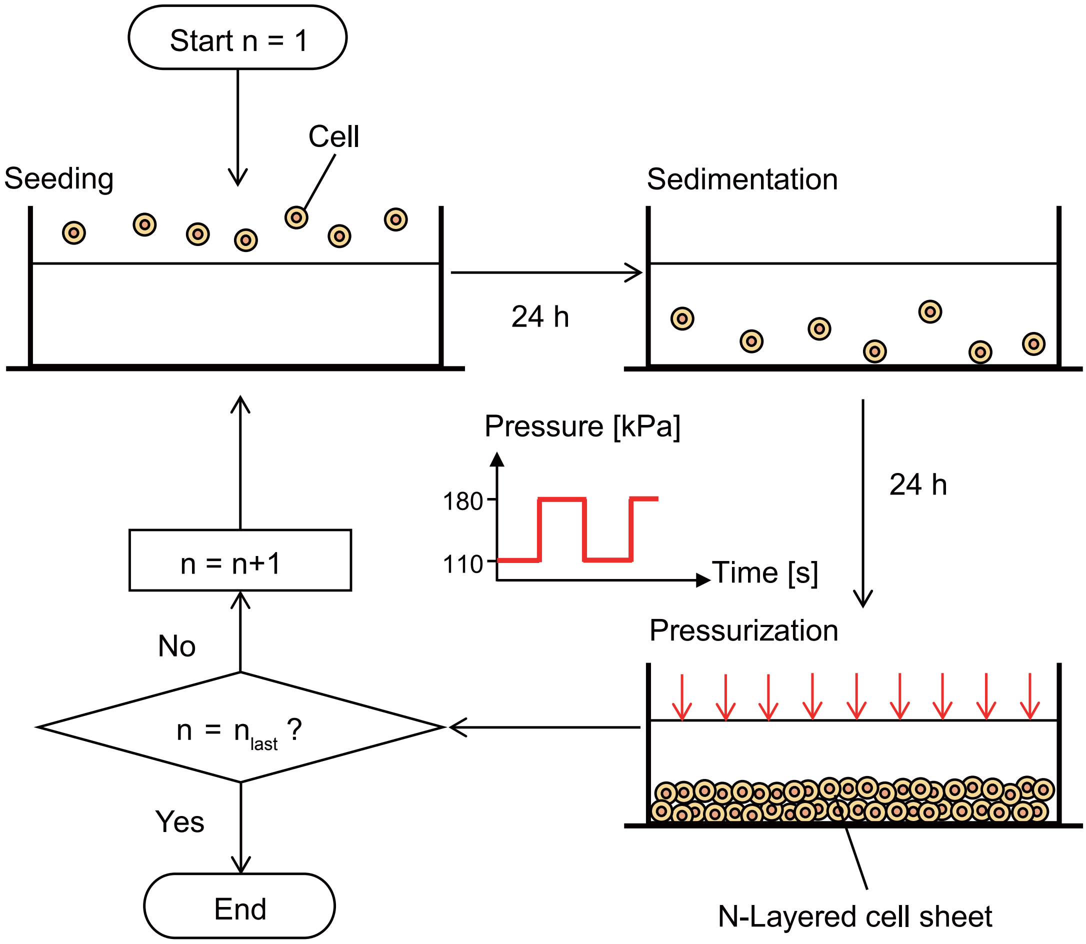

Supplemental Figure 4

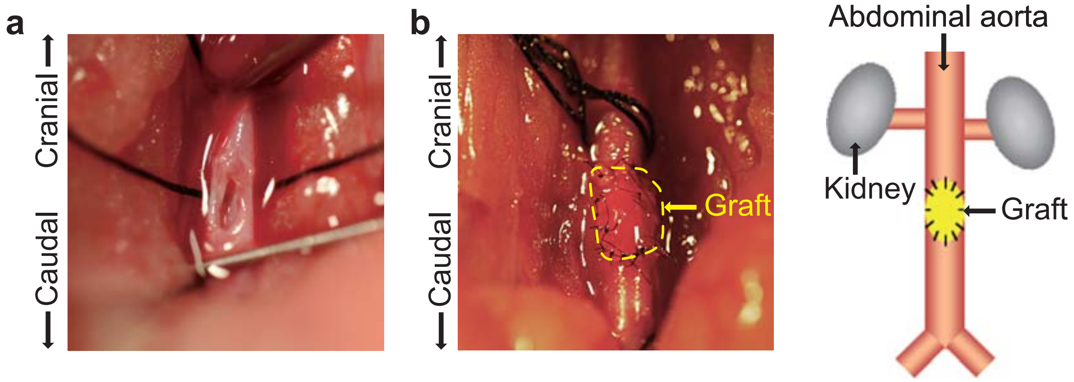

Supplemental Figure 5

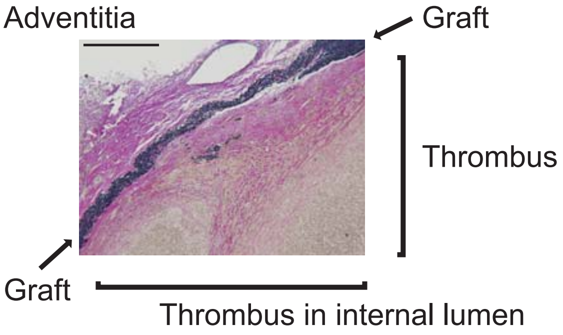

Supplement: Supplementary file 3 — Supplementary information [file 41598_2017_237_MOESM3_ESM.pdf]
